# Etymologia: *Escherichia coli*

**DOI:** 10.3201/eid2108.ET2108

**Published:** 2015-08

**Keywords:** etymologia, Escherichia coli, bacteria, colon bacillus, Theodor Escherich

## *Escherichia coli* [eshʺə-rikʹe-ə coʹlī]

A gram-negative, facultatively anaerobic rod, *Escherichia coli* was named for Theodor Escherich, a German-Austrian pediatrician. Escherich isolated a variety of bacteria from infant fecal samples by using his own anaerobic culture methods and Hans Christian Gram’s new staining technique. Escherich originally named the common colon bacillus *Bacterium coli commune*. Castellani and Chalmers proposed the name *E. coli* in 1919, but it was not officially recognized until 1958.
